# Video Surveillance of Hand Hygiene: A Better Tool for Monitoring and Ensuring Hand Hygiene Adherence

**DOI:** 10.5005/jp-journals-10071-23165

**Published:** 2019-05

**Authors:** Shruti Sharma, Vipul Khandelwal, Gajendra Mishra

**Affiliations:** 1,3 Department of Microbiology, Apex Hospital, Jaipur, Rajasthan, India; 2 Department of Internal Medicine and Critical Care, Apex Hospital, Jaipur, Rajasthan, India

**Keywords:** Compliance monitoring, Hand hygiene, Video surveillance, WHO five key moments

## Abstract

**Introduction:**

Hand hygiene practice, as correctly said, is the backbone of infection control and it has been proven to limit infections in hospital settings. Currently most healthcare facilities monitor hand hygiene compliance by direct observation technique.

We decided to use video surveillance as a tool to monitor hand hygiene compliance and its impact.

**Materials and Methods:**

This study was conducted over a period of 6 months from March 2018 to August 2018 at Apex Hospital, Jaipur, India.

We compared direct observation of ICU, High Dependency Units, and Emergency with video surveillance in these areas.

**Results and Observations:**

In this study, direct observation and video audit were compared from March 2018 to August 2018. During March to August, average compliance rates of direct observation and video surveillance were compared. In month of march, they were 67% and 20%, respectively and in the month of august, they were 81% and 47%, respectively.

**Conclusion:**

In our study, We can conclude in our study that video monitoring combined with direct observation can produce a significant and sustained improvement in hand hygiene compliance and can improve quality of patient care.

**How to cite this article:**

Sharma S, Khandelwal V, Mishra G. Video Surveillance of Hand Hygiene: A Better Tool for Monitoring and Ensuring Hand Hygiene Adherence. Indian J Crit Care Med 2019;23(5):224–226.

## INTRODUCTION

Hand hygiene practice, as correctly said, is the backbone of infection control and it has been proven to limit infections in hospital settings.^1^ One of the most important component of infection control program is to monitor hand hygiene compliance.^2,3^ WHO recommends regular hand hygiene compliance monitoring to improve the hand hygiene compliance. WHO recommends five key moments of hand hygiene, these are:

- Before touching a patient
- Before clean/aseptic procedures
- After body fluid exposure/risk
- After touching a patient
- After touching patient's surroundings^4,5^

Currently most healthcare facilities monitor hand hygiene compliance by direct observation technique, as this is considered “gold standard”.^6^ But this approach has its own limitations. Direct observation technique is most of the time affected by observer and other kind of biases, which can influence the action of the person being observed and sometimes does not give us the actual data of hand hygiene compliance.^6–9^ It is observed that direct observation gives us false high results than actual hand hygiene compliance. Furthermore, we cannot rely solely on direct observation technique for hand hygiene compliance monitoring as it has sampling bias also^6^ and sometimes the compliance vary from 4 to 100%.^4^ Video surveillance for compliance monitoring had been observed in many different industries like sports etc., as well as in hospital settings too for different purposes.^11^ Some studies have used video monitoring for hand hygiene monitoring as well.^12,13^ We also decided to use video surveillance as a tool to monitor hand hygiene compliance and its impact.

## MATERIALS AND METHODS

This study was conducted over a period of 6 months from March 2018 to August 2018 at Apex Hospital, Jaipur, India. Previously, we were using direct observation technique as the sole monitoring tool for hand hygiene compliance. We gave regular training for hand hygiene as before. No extra training was done in the study period.

For hand hygiene compliance monitoring, we used following formula:


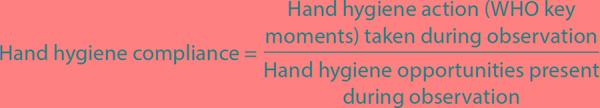


We compared direct observation of ICU, high dependency unit (HDU), and emergency (ER) with video surveillance in these areas. Direct observation was done for 30 minutes in each area, cumulatively 4 hours/day. From March onward, video surveillance was introduced for hand hygiene compliance monitoring and it was prior informed to all doctors and staff. Video surveillance was also done for the same duration i.e. 30 minutes. During video surveillance, no observer was physically present in those areas.

### RESULTS AND OBSERVATIONS

In this study, direct observation and video audit were compared from March 2018 to August 2018 between doctors, nurses, and housekeeping staff (Tables 1 to 6).

**Fig. 1:**
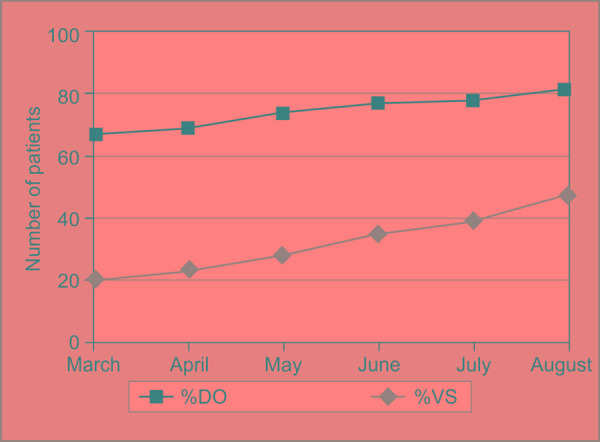
Compliance of hand hygiene according to direct observation (DO) and video surveillance (VS)

During March to August, average compliance rates of direct observation and video surveillance were compared. In month of march, they were 67% and 20%, respectively and in the month of august, they were 81% and 47%, respectively (Fig. 1).

## DISCUSSION

In our study, we observed WHO five key moments of hand hygiene in our hand hygiene monitoring. This study demonstrates that the hand hygiene compliance rate by direct observation technique and by video surveillance showed significant difference at the starting of study^7,12,14–18^ but this difference started to reduce later in the study, though not completely.^12,13^

Direct observation technique can have a disadvantage of observer bias, which can be due to multiple factors.^7,15–17^ The study of Armellino and colleagues showed reduced selection bias in video surveillance in comparison to direct observation that falsely increased rates due to Hawthorene effect or observer effect.^12,13^

**Table 1:** Comparison of direct observation vs video surveillance (March)

		*% (DO)*	*% (VS)*
ICU	Doctors	72	20
	Nursing staff	72	21
	Housekeeping staff	61	15
HDU	Doctors	68	20
	Nursing staff	71	22
	Housekeeping staff	60	17
Emergency	Doctors	70	22
	Nursing staff	68	23
	Housekeeping staff	64	18

**Table 2:** Comparison of direct observation vs video surveillance (April)

		*% (DO)*	*% (VS)*
ICU	Doctors	71	25
	Nursing staff	76	25
	Housekeeping staff	62	17
HDU	Doctors	68	23
	Nursing staff	71	25
	Housekeeping staff	60	18
Emergency	Doctors	70	28
	Nursing staff	68	29
	Housekeeping staff	64	18

**Table 3:** Comparison of direct observation vs video surveillance (May)

		*% (DO)*	*%(VS)*
ICU	Doctors	78	30
	Nursing staff	80	33
	Housekeeping staff	68	22
HDU	Doctors	76	29
	Nursing staff	79	30
	Housekeeping staff	65	20
Emergency	Doctors	75	32
	Nursing staff	78	35
	Housekeeping staff	65	21

**Table 4:** Comparison of direct observation vs video surveillance (June)

		*% (DO)*	*% (VS)*
ICU	Doctors	81	38
	Nursing staff	82	39
	Housekeeping staff	71	30
HDU	Doctors	79	37
	Nursing staff	80	35
	Housekeeping staff	67	29
Emergency	Doctors	79	38
	Nursing staff	82	38
	Housekeeping staff	69	29

**Table 5:** Comparison of direct observation vs video surveillance (July)

		*% (DO)*	*% (VS)*
ICU	Doctors	82	42
	Nursing staff	81	45
	Housekeeping staff	72	38
HDU	Doctors	80	40
	Nursing staff	81	39
	Housekeeping staff	70	37
Emergency	Doctors	82	39
	Nursing staff	83	38
	Housekeeping staff	70	35

**Table 6:** Comparison of direct observation vs video surveillance (August)

		*% (DO)*	*% (VS)*
ICU	Doctors	85	50
	Nursing Staff	83	50
	Housekeeping Staff	75	45
HDU	Doctors	84	48
	Nursing Staff	83	50
	Housekeeping Staff	74	42
Emergency	Doctors	85	48
	Nursing Staff	84	49
	Housekeeping Staff	74	40

We observed improved hand hygiene compliance overall, not just in presence of observer or camera.^12,13^ Staff was previously aware of the ongoing video surveillance but significant improvement was seen in subsequent months when feedback was given in monthly infection control meetings where difference in performance metrics between direct and video surveillance monitoring were displayed.

Although the purpose of this study was to observe hand hygiene compliance monitoring by video surveillance, we saw improvement in other areas of infection control practices, such as, standard precaution, aseptic technique during procedures etc. Employee privacy was maintained during the surveillance. Video tapes have been archived and can be further analyzed, which is the additional advantage of video monitoring.

We can conclude in our study that video monitoring combined with direct observation can produce a significant and sustained improvement in hand hygiene compliance and can improve quality of patient care.
